# A Limited-Memory BFGS Algorithm Based on a Trust-Region Quadratic Model for Large-Scale Nonlinear Equations

**DOI:** 10.1371/journal.pone.0120993

**Published:** 2015-05-07

**Authors:** Yong Li, Gonglin Yuan, Zengxin Wei

**Affiliations:** 1 College of Mathematics and Information Science, Guangxi University, Nanning, Guangxi, P.R.China; 2 Department of Mathematics, Baise University, Baise, Guangxi, P. R. China; Hangzhou Normal University, CHINA

## Abstract

In this paper, a trust-region algorithm is proposed for large-scale nonlinear equations, where the limited-memory BFGS (L-M-BFGS) update matrix is used in the trust-region subproblem to improve the effectiveness of the algorithm for large-scale problems. The global convergence of the presented method is established under suitable conditions. The numerical results of the test problems show that the method is competitive with the norm method.

## Introduction

Consider

$$\begin{array}{c} b\left(x\right)=0,\;\;x\in {ℜ}^{n}, \end{array}$$ (1)

where *b*:ℜ^*n*^ → ℜ^*n*^ is a system of nonlinear equations and *n* denotes the large-scale dimension. Problems involving non-linear equations can be found in numerous applications in engineering, such as nonlinear fitting, function approximating, parameter estimating and leaning models [1–5]. Large-scale nonlinear equations are believed to be difficult to solve because the relations with *x* are complex and because the dimension is high. Currently, many effective algorithms exist for addressing (1), such as the trust-region method [6–11], Levenberg-Marquardt method [12–14], quasi-Newton method [15–19], and Gauss-Newton method [20–25]. Let *θ* be the norm function defined by $\theta (x)=\frac{1}{2}{\left\|b(x)\right\|}^{2}$, and suppose that *b*(*x*) has a zero point. Then, (1) is equivalent to the following global optimization problem:

$$\begin{array}{c} \min\theta \left(x\right),\;\;x\in {ℜ}^{n}. \end{array}$$ (2)

In this paper, we will focus on the solution of (2) via the trust-region (TR) methods. The traditional TR methods, at each iterative point *x*_*k*_, obtain the trial step *d*_*k*_ using the following TR subproblem model:

$$\begin{array}{c} \min\;\;p_{k}\left(d\right)=\frac{1}{2}{∥b\left(x_{k}\right)+\nabla b\left(x_{k}\right)d∥}^{2}\;\;such\;\;that\;\;∥d∥\leq \Delta , \end{array}$$ (3)

where △ > 0 is the so-called TR radius and ‖⋅‖ denotes the Euclidean norm of vectors or their induced matrix. The TR method is very useful when the exact Jacobian or Hessian computation is inexpensive or possible. However, this case is rare in practice. It is not difficult to see that the Jacobian matrix ∇*b*(*x*) at every iteration must be computed, which obviously increases the workload and CPU time. Moreover, the global and superlinear convergence of the TR methods often requires that the nondegenerate assumption about ∇*b*(x*) holds, where x* is a solution of (1) and nondegeneracy means nonsingularity. The nondegeneracy of ∇*b*(x*) seems to be too stringent of a requirement for the purpose of ensuring the global and superlinear convergence. To overcome the above drawbacks, Yuan et al. [9] presented a new TR subproblem model defined by

$$\begin{array}{c} \min\;\;q_{k}\left(d\right)=\frac{1}{2}{∥b\left(x_{k}\right)+B_{k}d∥}^{2}\;\;such\;\;that\;\;∥d∥\leq \Delta_{k}, \end{array}$$ (4)

where the TR radius △_*k*_ = *c* ^*p*^‖*b*(*x*_*k*_)‖, *c* ∈ (0, 1), *p* is a nonnegative integer and *B*_*k*_ is generated by the following BFGS formula:

$$\begin{array}{c} B_{k+1}=B_{k}-\frac{B_{k}s_{k}s_{k}^{T}B_{k}}{s_{k}^{T}B_{k}s_{k}}+\frac{y_{k}{y_{k}}^{T}}{{y_{k}}^{T}s_{k}}, \end{array}$$ (5)

where *s*_*k*_ = *x*_*k*+1_ − *x*_*k*_, *y*_*k*_ = *b*(*x*_*k*+1_) − *b*(*x*_*k*_), *x*_*k*+1_ is the next iteration and *B*_0_ is an initial symmetric, positive definite matrix. They established the global and superlinear convergence without nondegeneracy. The numerical results show that the given algorithm is competitive with the normal TR method up to a dimension of 600. The subproblem (4) can be also rewritten as follows

$$\begin{array}{ccc} \min_{d\in {ℜ}^{n}}q_{k}\left(d\right) & = & \frac{1}{2}{∥b\left(x_{k}\right)+\nabla b\left(x_{k}\right)d∥}^{2}\\ & = & \frac{1}{2}{∥b(x_{k})∥}^{2}+b{\left(x_{k}\right)}^{T}\nabla b\left(x_{k}\right)d+\frac{1}{2}d^{T}\nabla b{\left(x_{k}\right)}^{T}\nabla b\left(x_{k}\right)d\\ & s.t. & ∥d∥\leq \Delta_{k}. \end{array}$$

Yuan et al. [8] consider the case with the symmetric Jacobian matrix ∇*b*(*x*_*k*_) and propose the following TR subproblem model:

$$\begin{array}{ccc} \min_{d\in {ℜ}^{n}}q_{k}\left(d\right) & = & \frac{1}{2}{∥b\left(x_{k}\right)+\nabla b\left(x_{k}\right)d∥}^{2}=\frac{1}{2}{∥b(x_{k})∥}^{2}+b{\left(x_{k}\right)}^{T}B_{k}d+\frac{1}{2}d^{T}B_{k}^{\prime }d\\ & s.t. & ∥d∥\leq \Delta_{k}, \end{array}$$

where $B_{k}^{\prime }$ is defined by the special BFGS update:

$$\begin{array}{c} B_{k+1}^{\prime }=B_{k}^{\prime }-\frac{B_{k}^{\prime }s_{k}s_{k}^{T}B_{k}^{\prime }}{s_{k}^{T}B_{k}^{\prime }s_{k}}+\frac{\delta_{k}{\delta_{k}}^{T}}{{\delta_{k}}^{T}s_{k}}, \end{array}$$ (6)

where *δ*_*k*_ = *b*(*x*_*k*_ + *y*_*k*_) − *b*(*x*_*k*_). About the above TR methods, the TR subproblems models will be repeatedly computed if the calculated trial step *d*_*k*_ does not satisfy the given conditions, which obviously increase the workload. To avoid this drawback, Yuan et al. [10] give the following TR model:

$$\begin{array}{ccc} \min_{d\in {ℜ}^{n}}q_{k}^{m}\left(d\right) & = & b{\left(x_{k}\right)}^{T}\nabla b\left(x_{k}\right)d+\frac{1}{2}d^{T}\nabla b{\left(x_{k}\right)}^{T}\nabla b\left(x_{k}\right)d\\ & s.t. & ∥\nabla b\left(x_{k}\right)d∥\leq \Delta_{k}^{m}, \end{array}$$

where ${△}_{k}^{m}=\max\{\|b(x_{k})\|,{△}_{k}\}.$ In this model, the next point is *x*_*k*+1_ = *x*_*k*_ + *d*_*k*_ if the trial step *d*_*k*_ satisfies the successful iteration; otherwise, the next point is *x*_*k*+1_ = *x*_*k*_ + *α*_*k*_ *d*_*k*_, where *α*_*k*_ > 0 is the so-called steplength designed by

$$\begin{array}{c} {∥b(x_{k}+\alpha_{k}d_{k})∥}^{2}\leq {∥b(x_{k})∥}^{2}+\delta \alpha_{k}^{2}b{\left(x_{k}\right)}^{T}d_{k}, \end{array}$$ (7)

where *δ* ∈ (0, 1) and the technique was proposed by Yuan and Lu [16]. However, the above model can not ensure that the number of searching *α*_*k*_ > 0 is small. Many TR model methods (see [7, 12] etc.) have been developed for solving nonlinear equations. However, these TR methods require the matrix information (the Jacobian matrix or the BFGS update matrix), which will increase the computational complexity. This observation motivate us find another approach to generate the matrix information requiring minimal memory.

Based on the problem (4), we will use the limited-memory BFGS (L-M-BFGS) method instead of the BFGS update because the former often provides a fast rate of linear convergence and requires minimal memory. The L-BFGS method is an adaptation of the standard BFGS method [26]. The implementation is identical to that of the normal BFGS method, the difference between the L-M-BFGS method and the BFGS method is that the inverse Hessian approximation is not explicitly formed, but defined by a small number of BFGS updates. At every iteration *x*_*k*_, the L-M-BFGS method stores a small number (say, *m*) of correction pairs {*s*_*i*_, *y*_*i*_}(*i* = *k* − 1, …, *k* − *m*) to obtain *H*_*k*+1_, instead of storing the matrices *H*_*k*_, where

$$\begin{array}{c} s_{k}=x_{k+1}-x_{k},\;\;\;\;\;y_{k}=b\left(x_{k+1}\right)-b\left(x_{k}\right), \end{array}$$

with *H*_*k*_ being the inverse of *B*_*k*_. The L-M-BFGS update formula is defined as

$$\begin{array}{ccc} H_{k+1} & = & V_{k}^{T}\left[V_{k-1}^{T}H_{k-1}V_{k-1}+\rho_{k-1}s_{k-1}s_{k-1}^{T}\right]V_{k}+\rho_{k}s_{k}s_{k}^{T}\\ & = & V_{k}^{T}V_{k-1}^{T}H_{k-1}V_{k-1}+V_{k}^{T}\rho_{k-1}s_{k-1}s_{k-1}^{T}V_{k}+\rho_{k}s_{k}s_{k}^{T}\\ & = & \cdots \\ & = & \left[V_{k}^{T}\cdots V_{k-m+1}^{T}\right]H_{k-m+1}\left[V_{k-m+1}\cdots V_{k}\right]+\rho_{k+m-1}[V_{k-1}^{T}\\ & \cdots & V_{k-m+2}^{T}]s_{k-m+1}s_{k-m+1}^{T}\left[V_{k-m+2}\ldots V_{k-1}\right]+\cdots +\rho_{k}s_{k}s_{k}^{T}, \end{array}$$ (8)

where $\rho_{k}=\frac{1}{y_{k}^{T}s_{k}}$ and $V_{k}=I-\rho_{k}y_{k}s_{k}^{T}$. It has been proved that the L-M-BFGS method is very suitable for large-scale systems of nonlinear equations [17]. Therefore, combining the L-M-BFGS update (8) and (4), we design the L-M-BFGS TR subproblem model as follows

$$\begin{array}{c} \min\;\;q_{k}\left(d\right)=\frac{1}{2}{∥b\left(x_{k}\right)+H_{k}^{-1}d∥}^{2}\;\;such\;\;that\;\;∥d∥\leq \Delta_{k}, \end{array}$$ (9)

where △_*k*_ = *c* ^*p*^‖*b*(*x*_*k*_)‖.

This paper is organized as follows. In the next section, the algorithm is discussed. In Section 3, the global convergence is established. Finally, the numerical results are reported in Section 4.

## The L-M-BFGS Trust-Region Method

The algorithm is given as follows.

- Algorithm 1.

**Initial:** Given the constants *ρ*, *c* ∈ (0, 1), *p* = 0, *ϵ* > 0, *x*_0_ ∈ ℜ^*n*^, *H*_0_ ∈ ℜ^*n*^ × ℜ^*n*^ is symmetric and positive definite. Set *k*: = 0;

**Step 1:** If ‖*b*_*k*_‖ < *ϵ*, stop;

**Step 2:** Solve the TR subproblem (9) with △ = △_*k*_ to obtain *d*_*k*_;

**Step 3:** Calculate the following ratio of the actual reduction over the predicted reduction:

$$\begin{array}{c} r_{k}^{p}=\frac{\theta \left(x_{k}+d_{k}\right)-\theta \left(x_{k}\right)}{q_{k}\left(d_{k}\right)-q_{k}\left(0\right)}. \end{array}$$ (1)

If $r_{k}^{p}<\rho ,$ then we let *p* = *p* + 1, go to Step 2. Otherwise, go to Step 4;

**Step 4:** Set *x*_*k*+1_ = *x*_*k*_ + *d*_*k*_, *y*_*k*_ = *b*_*k*+1_ − *b*_*k*_. Use (8) to generate the updated matrix *H*_*k*+1_ with positive definiteness.

**Step 5:** Let *k* ≔ *k* + 1, *p* = 0, and go to Step 1.

**Remark 1.** (i) In Algorithm 1, the “Step 2-Step 3-Step 2” procedure is called the inner cycle.

(ii) It is well known that the positive definiteness of the update matrix *H*_*k*_ is very important when analyzing the convergence of the algorithm. Byrd et al. [27] showed that the limited-memory BFGS matrix has this property if the curvature (*s*_*k*_)^*T*^ *y*_*k*_ > 0 is satisfied. Similarly, Powell [28] proposed that *y*_*k*_ should be as follows:

$$\begin{array}{c} y_{k}=\{\begin{array}{cc} y_{k}, & \;\;\text{if}\;\;s_{k}^{T}y_{k}\geq 0.2s_{k}^{T}B_{k}s_{k},\\ \theta_{k}y_{k}+\left(1-\theta_{k}\right)B_{k}s_{k}, & \;\;\;\text{otherwise}, \end{array} \end{array}$$

where $\theta_{k}=\frac{0.8s_{k}^{T}B_{k}s_{k}}{s_{k}^{T}B_{k}s_{k}-s_{k}^{T}y_{k}},$ *B*_*k*_ is an approximation of ∇*b*(*x*_*k*_) and $B_{k}=H_{k}^{-1}.$ Therefore, Step 4 of Algorithm 1 is reasonable.

To compare with the normal TR method, another algorithm is given and we call it Algorithm 2.

- Algorithm 2.(BFGS Trust-Region Method)

**Step 2:** Solve the TR subproblem (4) with △ = △_*k*_ to obtain *d*_*k*_;

**Step 4:** Set *x*_*k*+1_ = *x*_*k*_ + *d*_*k*_, *y*_*k*_ = *b*_*k*+1_ − *b*_*k*_. Use (5) to generate the updated matrix $H_{k+1}=B_{k+1}^{-1}$ with positive definiteness.

**Remark 2.** It is easy to see that the difficult step of these two algorithms is the Step 2. The following *Dogleg* method [29] is used to solve the TR subproblem models (4) and (9) to obtain *d*_*k*_.

- At the *k*th iteration:

set *ϖ*_*k*_ = *H*_*k*_ *b*(*x*_*k*_);

If ‖*ϖ*_*k*_‖ ≤ △_*k*_, let *d*_*k*_ = −*ϖ*_*k*_; Otherwise, set $\varsigma_{k}=\frac{{\left\|H_{k}^{-1}b(x_{k})\right\|}^{2}}{b{\left(x_{k}\right)}^{T}H_{k}^{-1}H_{k}^{-1}H_{k}^{-1}b(x_{k})},\;\;\varrho_{k}=-\varsigma_{k}H_{k}^{-1}b(x_{k}),\;\;\tau_{k}=-\varpi_{k}:$

If ς_*k*_ ≥ 0 and ς_*k*_ ≤ 1 hold, set *d*_*k*_ = ς_*k*_ϱ_*k*_; If ς_*k*_ ≥ 1 and ς_*k*_ ≤ 2 hold, set *d*_*k*_ = ϱ_*k*_ + (ς_*k*_ − 1)(*τ*_*k*_ − ϱ_*k*_).

## Convergence Analysis

This section will provide some convergence results under the following assumptions.

**Assumption A** **(i)** Let the level set Ω

$$\begin{array}{c} \Omega =\{x|\theta \left(x\right)\leq \theta \left(x_{0}\right)\} \end{array}$$ (1)

be bounded, and let *b*(*x*) be twice continuously differentiable on an open convex set Ω_1_ containing Ω.

**(ii)** The relation

$$\begin{array}{c} ∥\left[\nabla b\left(x_{k}\right)-H_{k}^{-1}\right]b_{k}∥=O(∥d_{k}∥) \end{array}$$ (2)

holds.

Assumption A(i) implies that there exists *M*_1_ > 0 such that

$$\begin{array}{c} ∥\nabla b{\left(x_{k}\right)}^{T}\nabla b\left(x_{k}\right)∥\leq M_{1},\;\forall \;\;k. \end{array}$$ (3)

Similar to Moré [30], Yuan et al. [9], and Yuan and Sun [11], we can obtain the following lemma.

**Lemma 0.1** *Let the predicted reduction be*

$$\begin{array}{c} Pred_{k}\left(d_{k}\right)=q_{k}\left(d_{k}\right)-q_{k}\left(0\right) \end{array}$$ (4)

*and let* *d*_*k*_ *be the solution of* (9). *Then, we have*

$$\begin{array}{c} Pred_{k}\left(d_{k}\right)\leq -\frac{1}{2}∥H_{k}^{-1}b_{k}∥\min\left\{\Delta_{k},\frac{∥H_{k}^{-1}b_{k}∥}{{∥H_{k}^{-1}∥}^{2}}\right\}. \end{array}$$ (5)

**Proof.** For any *α* ∈ [0, 1], by the property of the solution of (9) named *d*_*k*_, we obtain

$$\begin{array}{ccc} Pred_{k}\left(d_{k}\right) & \leq & Pred_{k}\left(-\alpha \frac{\Delta_{k}}{∥H_{k}^{-1}b_{k}∥}H_{k}^{-1}b_{k}\right)\\ & = & \frac{1}{2}\alpha^{2}\Delta_{k}^{2}{\left(H_{k}^{-1}H_{k}^{-1}b_{k}\right)}^{T}\left(H_{k}^{-1}H_{k}^{-1}b_{k}\right)/{∥H_{k}^{-1}b_{k}∥}^{2}-\alpha \Delta_{k}∥H_{k}^{-1}b_{k}∥\\ & \leq & \frac{1}{2}\alpha^{2}\Delta_{k}^{2}∥H_{k}^{-1}H_{k}^{-1}∥-\alpha \Delta_{k}∥H_{k}^{-1}b_{k}∥. \end{array}$$

Thus, the inequality

$$\begin{array}{c} -Pred_{k}\left(d_{k}\right)\geq \max_{0\leq \alpha \leq 1}[\alpha \Delta_{k}∥H_{k}^{-1}b_{k}∥-\frac{1}{2}\alpha^{2}\Delta_{k}^{2}{∥H_{k}^{-1}∥}^{2}]\geq \frac{1}{2}∥H_{k}^{-1}b_{k}∥\min\left\{\Delta_{k},\frac{∥H_{k}^{-1}b_{k}∥}{{∥H_{k}^{-1}∥}^{2}}\right\} \end{array}$$

holds. This completes the proof.

**Lemma 0.2** *Let* *d*_*k*_ *be the solution of* (9), *and let the actual reduction* *Ared*_*k*_(*d*_*k*_) *be*

$$\begin{array}{c} Ared_{k}\left(d_{k}\right)=\theta \left(x_{k}+d_{k}\right)-\theta \left(x_{k}\right). \end{array}$$ (6)

*Suppose that Assumption A holds and that the sequence* {*x*_*k*_, *d*_*k*_} *is generated by Algorithm 1. Then, the inner cycle of Algorithm 1 will stop in a finite number of steps*.

**Proof.** We will establish this lemma by contradiction. Suppose that the inner cycle of Algorithm 1 infinitely circles at *x*_*k*_, namely, *p* → ∞, $r_{k}^{p}<\rho$ and *c* ^*p*^ → 0 hold. It is easy to obtain ‖*b*_*k*_‖ ≥ *ϵ*, or the algorithm stops. Thus, we can conclude that ‖*d*_*k*_‖ ≤ △_*k*_ = *c* ^*p*^‖*b*_*k*_‖ → 0 is true.

By the definitions of *d*_*k*_, *Ared*_*k*_(*d*_*k*_), *Pred*_*k*_(*d*_*k*_), and Assumption A, we obtain

$$\begin{array}{ccc} |Ared_{k}\left(d_{k}\right)-Pred_{k}\left(d_{k}\right)| & = & |\theta \left(x_{k}+d_{k}\right)-q_{k}\left(d_{k}\right)|\\ & = & \frac{1}{2}|{∥b_{k}+\nabla b\left(x_{k}\right)d_{k}+O\left({∥d_{k}∥}^{2}\right)∥}^{2}-{∥b_{k}+H_{k}^{-1}d_{k}∥}^{2}|\\ & = & |b_{k}^{T}\nabla b\left(x_{k}\right)d_{k}-b_{k}^{T}H_{k}^{-1}d_{k}+O\left({∥d_{k}∥}^{2}\right)+O\left({∥d_{k}∥}^{4}\right)|\\ & \leq & ∥\left[\nabla b\left(x_{k}\right)-H_{k}^{-1}\right]b_{k}∥∥d_{k}∥+O\left({∥d_{k}∥}^{2}\right)+O\left({∥d_{k}∥}^{4}\right)\\ & = & O({∥d_{k}∥}^{2}). \end{array}$$ (7)

where the second equality follows Taylor’s formula. It follows from Lemma 0.1 and 7) that

$$\begin{array}{c} |r_{k}^{p}-1|=\frac{|Ared_{k}\left(d_{k}\right)-Pred_{k}\left(d_{k}\right)|}{|Pred_{k}\left(d_{k}\right)|}\leq \frac{2O({∥d_{k}∥}^{2})}{\Delta_{k}∥B_{k}b_{k}∥}\to 0. \end{array}$$

Thus, if *p* is sufficiently large, we have $r_{k}^{p}\geq \rho .$ This relation contradicts $r_{k}^{p}<\rho .$ The proof is complete.

The above lemma shows that the given algorithm is well-defined.

**Theorem 0.1** *Let Assumption A hold, and let* {*x*_*k*_} *be generated by Algorithm 1. Then*, {*x*_*k*_} ⊂ Ω, *and* {*θ*(*x*_*k*_)} *converges. In particular, Algorithm 1 either stops in a finite number of steps or generates an infinite sequence* {*x*_*k*_} *such that*

$$\begin{array}{c} \lim_{k\to \infty }∥b_{k}∥=0. \end{array}$$ (8)

**Proof.** By the definition of Algorithm 1, we obtain $r_{k}^{p}\geq \rho >0.$ Combining this with Lemma 0.1 generates

$$\begin{array}{c} \theta \left(x_{k+1}\right)\leq \theta \left(x_{k}\right)\leq \cdots \leq \theta \left(x_{0}\right). \end{array}$$ (9)

Thus, {*x*_*k*_} ⊂ Ω holds. Considering *θ*(*x*_*k*_) ≥ 0, we then conclude that {*θ*(*x*_*k*_)} converges.

If Algorithm 1 does not stop after a finite number of steps, again by (9) and *θ*(*x*_*k*_) ≥ 0, we easily conclude that

$$\begin{array}{c} \theta (x_{k})\to 0,\;\;k\to \infty , \end{array}$$

which shows that *b*(*x*_*k*_) → 0, *k* → ∞. The proof is complete.

Theorem 0.1 proves that the sequence {*x*_*k*_} of Algorithm 1 converges such that ‖*b*(*x*_*k*_)‖ → 0 without the assumption that ∇*b*(x*) is nondegenerate, where x* is a cluster point of {*x*_*k*_}.

## Numerical Results

This section will report numerical results obtained using Algorithm 1. The test functions with initial points *x*_0_ are listed as follows:

$$\begin{array}{c} b\left(x\right)={\left(f_{1}\left(x\right),f_{2}\left(x\right),\cdots ,f_{n}\left(x\right)\right)}^{T}. \end{array}$$

**Function 1.** Exponential function 2

$$\begin{array}{ccc} f_{1}\left(x\right) & = & e^{x_{1}}-1,\\ f_{i}\left(x\right) & = & \frac{i}{10}\left(e^{x_{i}}+x_{i-1}-1\right),\;\;i=2,3,\cdots ,n \end{array}$$

Initial guess: $x_{0}={\left(\frac{1}{n},\frac{1}{n},\cdots ,\frac{1}{n}\right)}^{T}.$

**Function 2.** Trigonometric function

$$\begin{array}{c} f_{i}\left(x\right)=2\left(n+i\left(1-\cos\left(x_{i}\right)\right)-\sin\left(x_{i}\right)-\sum_{k=1}^{n}\cos\left(x_{k}\right)\right)\left(2\sin\left(x_{i}\right)-\cos\left(x_{i}\right)\right),\;\;i=1,2,\cdots ,n \end{array}$$

Initial guess: $x_{0}={\left(\frac{101}{100n},\frac{101}{100n},\cdots ,\frac{101}{100n}\right)}^{T}.$

**Function 3.** Singular function

$$\begin{array}{ccc} f_{1}\left(x\right) & = & \frac{1}{3}x_{1}^{3}+\frac{1}{2}x_{2}^{2},\\ f_{i}\left(x\right) & = & -\frac{1}{2}x_{i}^{2}+\frac{i}{3}x_{i}^{3}+\frac{1}{2}x_{i+1}^{2},\;\;i=2,3,\cdots ,n-1,\\ f_{n}\left(x\right) & = & -\frac{1}{2}x_{n}^{2}+\frac{n}{3}x_{n}^{3}. \end{array}$$

Initial guess: *x*_0_ = (1, 1, ⋯, 1)^*T*^.

**Function 4.** Logarithmic function

$$\begin{array}{c} f_{i}\left(x\right)=\ln\left(x_{i}+1\right)-\frac{x_{i}}{n},\;\;i=1,2,3,\cdots ,n. \end{array}$$

Initial guess: *x*_0_ = (1, 1, ⋯, 1)^*T*^.

**Function 5.** Broyden Tridiagonal function [[31], pp. 471–472]

$$\begin{array}{ccc} f_{1}\left(x\right) & = & \left(3-0.5x_{1}\right)x_{1}-2x_{2}+1,\\ f_{i}\left(x\right) & = & \left(3-0.5x_{i}\right)x_{i}-x_{i-1}+2x_{i+1}+1,\;\;i=2,3,\cdots ,n-1,\\ f_{n}\left(x\right) & = & \left(3-0.5x_{n}\right)x_{n}-x_{n-1}+1. \end{array}$$

Initial guess: *x*_0_ = (−1, −1, ⋯, −1)^*T*^.

**Function 6.** Trigexp function

$$\begin{array}{ccc} f_{1}\left(x\right) & = & 3x_{1}^{3}+2x_{2}-5+\sin\left(x_{1}-x_{2}\right)\sin\left(x_{1}+x_{2}\right),\\ f_{i}\left(x\right) & = & -x_{i-1}e^{x_{i-1}-x_{i}}+x_{i}\left(4+3x_{i}^{2}\right)+2x_{i+1}\\ & + & \sin\left(x_{i}-x_{i+1}\right)\sin\left(x_{i}+x_{i+1}\right),\;\;i=2,3,\cdots ,n-1,\\ f_{n}\left(x\right) & = & -x_{n}e^{x_{n-1}-x_{n}}+4x_{n}-3. \end{array}$$

Initial guess: *x*_0_ = (0, 0, ⋯, 0)^*T*^.

**Function 7.** Strictly convex function 1 [[32], p. 29]

*b*(*x*) is the gradient of $h(x)=\sum_{i=1}^{n}(e^{x_{i}}-x_{i}).$

$$\begin{array}{c} f_{i}\left(x\right)=e^{x_{i}}-1,\;\;i=1,2,3,\cdots ,n \end{array}$$

Initial guess: $x_{0}={\left(\frac{1}{n},\frac{2}{n},\cdots ,1\right)}^{T}.$

**Function 8.** Variable dimensional function

$$\begin{array}{ccc} f_{i}\left(x\right) & = & x_{i}-1,\;\;i=1,2,3,\cdots ,n-2,\\ f_{n-1}\left(x\right) & = & \sum_{j=1}^{n-2}j\left(x_{j}-1\right),\\ f_{n}\left(x\right) & = & {\left(\sum_{j=1}^{n-2}j\left(x_{j}-1\right)\right)}^{2}. \end{array}$$

Initial guess: $x_{0}={\left(1-\frac{1}{n},1-\frac{2}{n},\cdots ,0\right)}^{T}.$

**Function 9.** Discrete boundary value problem [33].

$$\begin{array}{ccc} f_{1}\left(x\right) & = & 2x_{1}+0.5h^{2}{\left(x_{1}+h\right)}^{3}-x_{2},\\ f_{i}\left(x\right) & = & 2x_{i}+0.5h^{2}{\left(x_{i}+hi\right)}^{3}-x_{i-1}+x_{i+1},\;\;i=2,3,\cdots ,n-1\\ f_{n}\left(x\right) & = & 2x_{n}+0.5h^{2}{\left(x_{n}+hn\right)}^{3}-x_{n-1},\\ h & = & \frac{1}{n+1}. \end{array}$$

Initial guess: *x*_0_ = (*h*(*h* − 1), *h*(2*h* − 1), ⋯, *h*(*nh* − 1)).

**Function 10.** The discretized two-point boundary value problem that is similar to the problem in [34]

$$\begin{array}{c} b\left(x\right)=Ax+\frac{1}{{\left(n+1\right)}^{2}}F\left(x\right)=0, \end{array}$$

when *A* is the *n* × *n* tridiagonal matrix given by

$$\begin{array}{c} A=[\begin{array}{cccccc} 8 & -1\\ -1 & 8 & -1\\ & -1 & 8 & -1\\ & & \ddots & \ddots & \ddots & \\ & & & \ddots & \ddots & -1\\ & & & & -1 & 8 \end{array}] \end{array}$$

and *F*(*x*) = (*F*_1_(*x*), *F*_2_(*x*), …, *F*_*n*_(*x*))^*T*^, with *F*_*i*_(*x*) = sin *x*_*i*_ − 1, *i* = 1, 2, …, *n*, and *x* = (50, 0, 50, 0, ⋯).

All codes for the experiments were written in MATLAB r2009a and run on a PC with an E5507@2.27 GHz CPU and 2.99 GB of memory using the Windows XP operating system. The parameters were set as *ρ* = 0.0001, *ϵ* = 10^−5^, *c* = 0.1, *γ* = 0.7, where *B*_0_ and *H*_0_ are unit matrices and *m* = 6. In the inner iterations of Algorithm 1 and Algorithm 2, the trial step will be accepted when *p* > 5. We also stop the program if the number of iterations is larger than one thousand or when *θ*(*x*) < 10^−5^. The columns of the tables have the following meaning:

Dim: the dimension of the problem.
NG: the number of norm function evaluations.
NI: the total number of iterations.
GN: the normal value of *θ*(*x*) when the program stops.
NaN: fails to find the final values of *θ*(*x*).

From Table 1, it is clear that the new algorithm performs quite well in terms of solving these problems and that the dimension does not have an obvious influence on the performance of the presented method. Algorithm 2 can also successfully solve some of these problems. However, Algorithm 2 fails to solve problems 3 and 6.

**Table 1:** Numerical results using Algorithms 1 and 2.

**Algorithm 1**				**Algorithm 2**	
Functions	Dim	NI/NG	GN	NI/NG	GN
1	800	0/1	8.348973e-006	0/1	8.348973e-006
	1000	0/1	6.676674e-006	0/1	6.676674e-006
	2000	0/1	3.335834e-006	0/1	3.335834e-00
2	800	7/18	1.766154e-006	8/19	4.764396e-006
	1000	7/18	1.578116e-006	8/19	4.228277e-006
	2000	6/17	8.507453e-006	8/19	2.609172e-006
3	800	82/108	5.997218e-006	1000/1716	3.453004e+018
	1000	68/89	8.159424e-006	1000/1636	3.372369e+019
	2000	95/152	8.892174e-006	1000/1701	1.757490e+022
4	800	5/6	6.440159e-008	5/6	6.440159e-008
	1000	5/6	7.954854e-008	5/6	7.954854e-008
	2000	5/6	1.553487e-007	5/6	1.553487e-007
5	800	61/67	9.546109e-006	47/83	7.073607e-006
	1000	58/64	9.424256e-006	35/66	5.881295e-006
	2000	61/67	9.412218e-006	44/75	8.483566e-006
6	800	62/73	6.368574e-008	6/22	NaN
	1000	59/75	1.493209e-007	6/22	NaN
	2000	71/92	2.616014e-007	6/22	NaN
7	800	6/7	1.665516e-009	5/6	1.718325e-006
	1000	6/7	2.070362e-009	5/6	2.139904e-006
	2000	6/7	4.094910e-009	5/6	4.247951e-006
8	800	1/2	0.000000e+000	1/2	0.000000e+000
	1000	1/2	0.000000e+000	1/2	0.000000e+000
	2000	1/2	0.000000e+000	1/2	0.000000e+000
9	800	2/3	3.679023e-006	4/10	1.336824e-006
	1000	2/3	2.358640e-006	4/10	9.011723e-007
	2000	2/3	5.917002e-007	2/3	3.637130e-006
10	800	15/21	1.212419e-007	32/40	6.985962e-006
	1000	15/21	1.391409e-007	34/42	5.778079e-006
	2000	15/21	1.824225e-007	33/44	5.520880e-006

To clearly demonstrate these algorithms’ efficiencies, a tool developed by Dolan and Moré [35] is used to analyze them. In this tool, the so-called (cumulative) distribution function ϱ_*s*_ is defined by

$$\begin{array}{c} \varrho_{s}\left(\kappa \right)=\frac{1}{n_{p}}size\left\{p\in P\;:\;\pi_{p,s}\leq \kappa \right\}, \end{array}$$

where *p* is a problem, *s* is its solver, *n*_*p*_ is the number of problems, *n*_*s*_ is the number of existing solvers, *P* is the test set, *κ* denotes the spending time or the iteration number, and *π*_*p*,*s*_ is the performance ratio given by

$$\begin{array}{c} \pi_{p,s}=\frac{\kappa_{p,s}}{\min\{\kappa_{p,s}\;:\;s\in S\}}, \end{array}$$

where *S* is the set of solvers. According to the above formulas and the rules given in [35], the performance profile plot of Table 1 is given in the following two figures.

**Fig 1:**
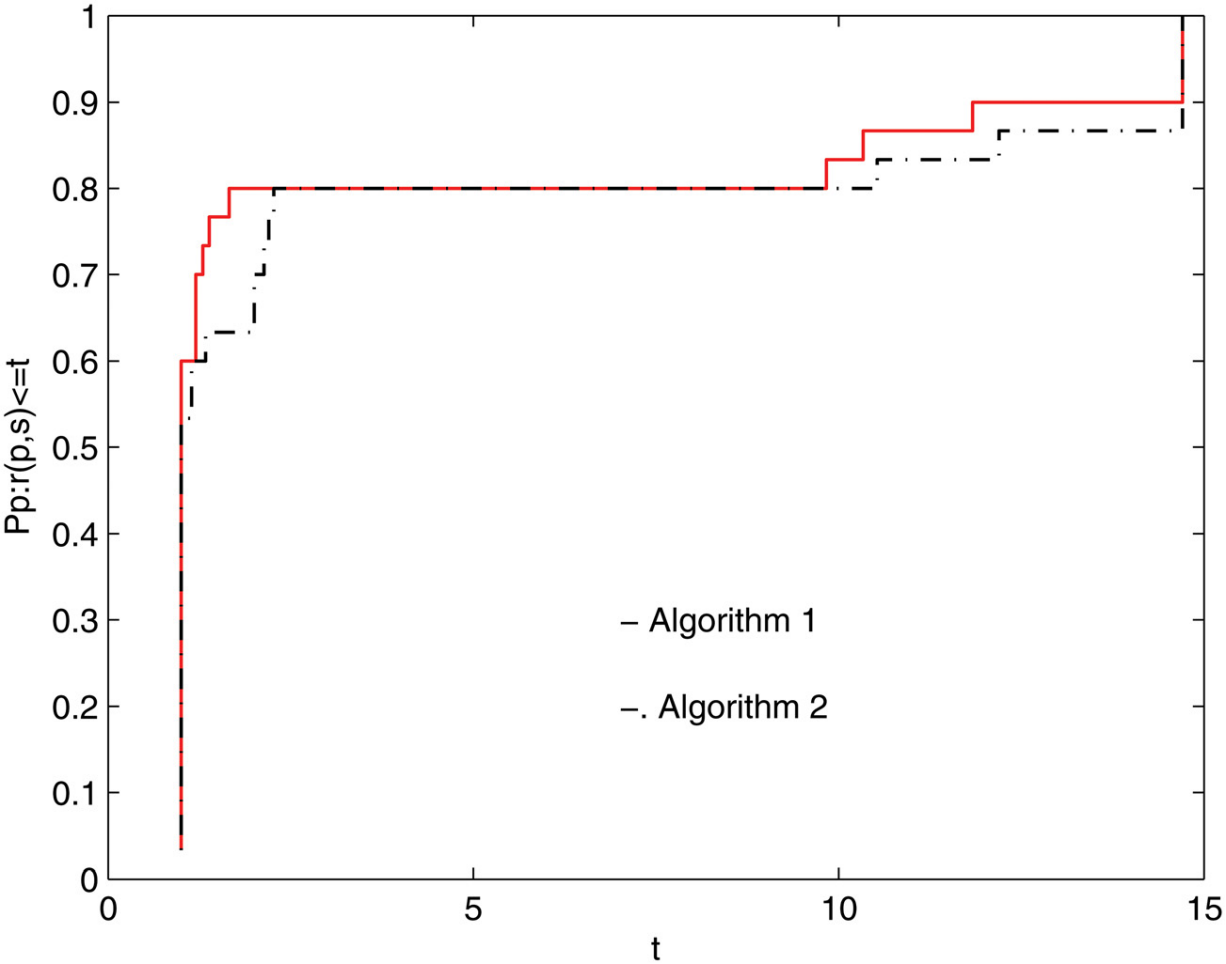
Performance profiles of these methods(NI).

**Fig 2:**
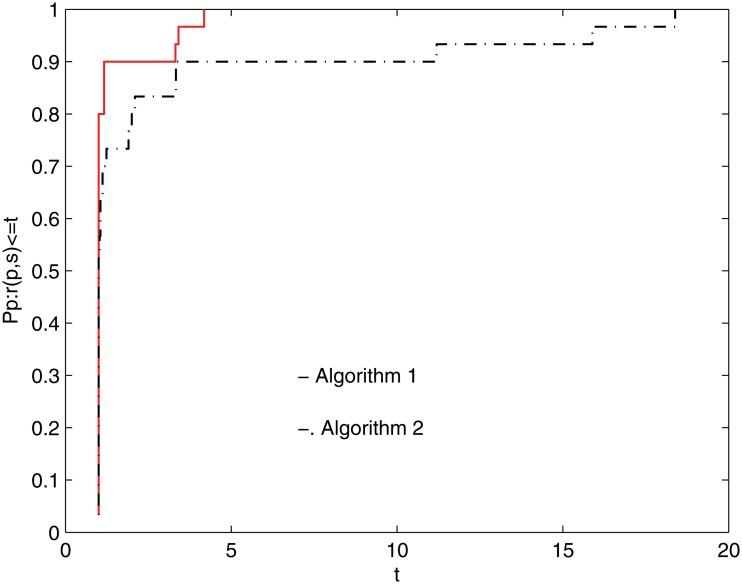
Performance profiles of these methods(NG).

Figs 1 and 2 show that the performance of these methods is a function of *NI* and *NG*, respectively, in Table 1. It is clear that the given method performs better because it has a higher probability of being the optimal solver. Fig 1 shows that Algorithm 1 is superior to Algorithm 2 for *κ* ≤ 14, approximately. From Fig 2, it is clear that the given method can successfully solve 100% of the test problems at *κ* ≈ 5 and that Algorithm 2 successfully solves the test problems at *κ* ≈ 17. Overall, the numerical results are interesting.

## Supporting Information:Legends of Figs 1 and 2

Dolan and Moré [35] developed a new tool to analyze the efficiency of algorithms. They introduced the notion of a performance profile for evaluating and comparing the performance of the set of solvers *S* on a test set *P*. Assuming that *n*_*s*_ solvers and *n*_*p*_ problems exist, for each problem *p* and solver *s*, they defined *κ*_*p*,*s*_ as the computing time (the number of function evaluations or some other metric) required to solve problem *p* using solver *s*.

Requiring a baseline for comparison, they compared the performance on problem *p* using solver *s* with the best performance by any solver on this problem, giving the the performance ratio

$$\begin{array}{c} \pi_{p,s}=\frac{\kappa_{p,s}}{\min\{\kappa_{p,s}\;:\;s\in S\}}. \end{array}$$

Suppose that a parameter *π*_*M*_ ≥ *π*_*p*,*s*_ for all *p*, *s* is chosen, and *π*_*p*,*s*_ = *π*_*M*_ if and only if solver *s* does not solve problem *p*.

The performance of solver *s* on any given problem might be of interest. More importantly, one would like to obtain an overall assessment of the performance of the solver. With this motivation, these authors defined

$$\begin{array}{c} \varrho_{s}\left(\kappa \right)=\frac{1}{n_{p}}size\left\{p\in P\;:\;\pi_{p,s}\leq \kappa \right\}. \end{array}$$

Specifically, ϱ_*s*_(*κ*) is the probability for solver *s* ∈ *S* that a performance ratio *π*_*p*,*s*_ is within a factor *κ* ∈ ℜ of the best possible ratio. Then, function ϱ_*s*_ is the (cumulative) distribution function for the performance ratio. The performance profile ϱ_*s*_ : ℜ ↦ [0, 1] for a solver is a nondecreasing, piecewise constant function and is continuous from the right at each breakpoint. The value of ϱ_*s*_(1) is the probability that the solver would outperform the remaining solvers.

According to the above rules, we know that one solver whose performance profile plot is on the top right outperforms the remaining solvers. Similar legends can be found in [10, 36–38].

## Conclusion

1. In this paper, a TR model combining with the L-M-BFGS technique is presented for nonlinear equations and the global convergence is established. Fact, this work is an extension of the paper [9] and the difference between these two papers is the update matrix: this paper chooses the L-M-BFGS formula and the paper [9] chooses the normal BFGS formula.
2. Since the L-M-BFGS technique requires minimal storage and is suitable for certain classes of large scale optimization problems, so we extend it to large scale nonlinear equations. Numerical results turn out the given algorithm is competitive to similar method for large-scale nonlinear equations.
3. It is well known that the conjugate gradient methods are effective techniques for large-scale optimization problems since they do not need the matrix information, which motivates us to use the conjugate gradient technique for large scale nonlinear equations. We think the numerical performance is also very interesting.
